# Iodine contrast exposure and incident COVID-19 infection

**DOI:** 10.3389/fmed.2022.1033601

**Published:** 2022-12-01

**Authors:** Karen Tsai, Kosuke Inoue, Michael McClean, Jonathan D. Kaunitz, Yasutada Akiba, Martin L. Lee, Natalia V. Neverova, Jesse W. Currier, Ramin Ebrahimi, Muhammad T. Bashir, Angela M. Leung

**Affiliations:** ^1^Endocrinology, Diabetes, and Metabolism Section, Medical Service, Veterans Affairs Greater Los Angeles Healthcare System, Los Angeles, CA, United States; ^2^Division of Endocrinology, Diabetes, and Metabolism, Department of Medicine, David Geffen School of Medicine, University of California, Los Angeles, Los Angeles, CA, United States; ^3^Department of Social Epidemiology, Graduate School of Medicine, Kyoto University, Kyoto, Japan; ^4^Department of Internal Medicine, Ito Hospital, Tokyo, Japan; ^5^Research Service, Veterans Affairs Greater Los Angeles Healthcare System, Los Angeles, CA, United States; ^6^Gastroenterology Section, Medical Service, Veterans Affairs Greater Los Angeles Healthcare System, Los Angeles, CA, United States; ^7^Division of Gastroenterology, Department of Medicine, David Geffen School of Medicine, University of California, Los Angeles, Los Angeles, CA, United States; ^8^Department of Surgery, David Geffen School of Medicine, University of California, Los Angeles, Los Angeles, CA, United States; ^9^Veterans Affairs Health Services Research and Development, Center for the Study of Health Care Innovation, Implementation, and Policy, Veterans Affairs Greater Los Angeles Healthcare System, Los Angeles, CA, United States; ^10^Department of Biostatistics, Fielding School of Public Health, University of California, Los Angeles, Los Angeles, CA, United States; ^11^Cardiology Section, Medical Service, Veterans Affairs Greater Los Angeles Healthcare System, Los Angeles, CA, United States; ^12^Division of Cardiology, Department of Medicine, David Geffen School of Medicine, University of California, Los Angeles, Los Angeles, CA, United States

**Keywords:** COVID-19, SARS-CoV-2, Veterans, iodine, iodinated contrast

## Abstract

**Background:**

Iodine and particularly its oxidated forms have long been recognized for its effective antiseptic properties. Limited *in vitro* and *in vivo* data suggest that iodine exposure may rapidly inactivate, reduce transmission, and reduce infectivity of SARS-CoV-2. We hypothesized that iodine exposure may be associated with decreased incident COVID-19 infection.

**Methods:**

A retrospective population-level cohort analysis was performed of the U.S. Veterans Health Administration between 1 March 2020 and 31 December 2020, before the widespread availability of vaccines against SARS-CoV-2. Multivariable logistic regression models estimated the adjusted odds ratios (OR) and 95% confidence intervals (CI) of the associations between iodinated contrast exposure and incident COVID-19 infection, adjusting for age, sex, race/ethnicity, place of residence, socioeconomic status, and insurance status.

**Results:**

530,942 COVID-19 tests from 333,841 Veterans (mean ± SD age, 62.7 ± 15.2 years; 90.2% men; 61.9% non-Hispanic Whites) were analyzed, of whom 9% had received iodinated contrast ≤60 days of a COVID-19 test. Iodine exposure was associated with decreased incident COVID-19 test positivity (OR, 0.75 95% CI, 0.71–0.78). In stratified analyses, the associations between iodinated contrast use and decreased COVID-19 infection risk did not differ by age, sex, and race/ethnicity.

**Conclusion:**

Iodine exposure may be protective against incident COVID-19 infection. Weighed against the risks of supraphysiologic iodine intake, dietary, and supplemental iodine nutrition not to exceed its Tolerable Upper Limit may confer an antimicrobial benefit against SARS-CoV-2. A safe but antimicrobial level of iodine supplementation may be considered in susceptible individuals, particularly in geographic regions where effective COVID-19 vaccines are not yet readily available.

## Introduction

COVID-19 is an multiorgan illness caused by Severe Acute Respiratory Syndrome-Coronavirus 2 (SARS-CoV-2) that was first identified in December 2019. On 11 March 2020, the World Health Organization (WHO) declared the associated COVID-19 infection a global pandemic, marking an unprecedented public health issue that has adversely impacted the health and wellbeing of humanity worldwide. Due to the far-reaching consequences of the COVID-19 pandemic, resulting in over 6 million deaths as of March 2022 (1), there has been substantial research regarding measures to reduce its transmission and reduce the morbidity and mortality associated with COVID-19 infection.

Iodine is an element and micronutrient that has long been recognized for its effective antiseptic properties (2–4). Physiologically, iodine is essential for thyroid hormone production, with 150 mcg iodine/day as the recommended intake in adults needed to ensure normal thyroid function (5). There are also potential risks of excess iodine exposure (6); indeed, transient or permanent iodine-induced hypothyroidism or hyperthyroidism may further lead to downstream adverse health effects, including atrial fibrillation, that may be fatal if left untreated. Since the advent of iodine contrast agents to enhance imaging visibility in the 1950s, there has been exponential growth in the utilization of these procedures. There has been a more than doubling of CT scans performed in the U.S. over the past two decades (56/1,000 person-years in 2,000 vs. 141/1,000 person-years in 2016) (7), and approximately 40% of CT scans worldwide are contrast-enhanced, with iodine as the predominant contrast medium of choice (8). A single dose of iodinated contrast administered for CT scanning, coronary angiography, and other radiologic procedures can contain up to 13,500 mcg of free iodine and 15–60 g of bound iodine, which is equivalent to more than several hundred times the recommended daily iodine intake (9). Following exposure to an iodinated contrast agent, iodine stores in the body remain elevated, providing a continuous pool of excess iodine for up to several months (10).

Given its antimicrobial properties, it is plausible that iodine exposure may modify the risk of SARS-CoV-2 infection. At the cellular level, the lactoperoxidase (LPO) dual oxidase (DUOX) halide (LDH) system converts the exogenous halide iodine and pseudohalide thiocyanate (SCN-), as taken up and concentrated by the sodium/iodide symporter (NIS) (11) expressed in the epithelial cells of the salivary glands and bronchial epithelium, into oxidant disinfectants such as hypoiodous acid (HOI) hypoiodite (OI-), and hypothiocyanite (OSCN-) that are related to the common disinfectant, hypochlorite (OCl-) (12–14). HOI/OI- and OSCN- have strong antimicrobial effects, especially against the enveloped viruses that include coronaviruses (15, 16). The location of the LDH system on surfaces topologically exposed to the environment (e.g., salivary glands, airway, and stomach) further underscore the importance of the LDH system as a robust innate microbial defense mechanism (17). *In vitro* data demonstrates that treatment of SARS-CoV-2 with iodine for 2 min decreases viral infectivity to undetectable levels, equivalent to the efficacy of 70% EtOH against COVID-19 (16, 18). Small pilot studies composed of up to 35 volunteers have shown that gargling of povidone iodine, a commonly used topical antiseptic to oral passageways, for as brief as 20 s can rapidly inactivate, reduce transmission, and reduce infectivity of SARS-CoV-2 (15, 16, 19–21).

Given the excessive iodine content in iodinated contrast media, its routine clinical use in healthcare offers an ideal setting to examine the antimicrobial effects of a high iodine load. In the current study, we analyzed a large healthcare database and hypothesize that prior iodinated contrast exposure reduces the incidence of SARS-CoV-2 infection.

## Materials and methods

### Study cohort

**FIGURE 1:**
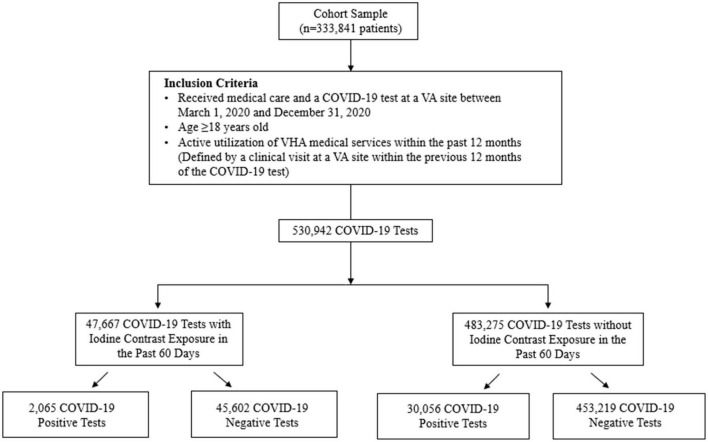
Flow of study sample selection.

A retrospective population-level cohort study was conducted of the Veterans Affairs (VA) Corporate Data Warehouse (CDW) database, the national repository of clinical and administrative records corresponding to all U.S. Veterans Health Administration (VHA) inpatient and outpatient sites, including the housing of the VA COVID-19 Shared Data Resource database. The cohort included patients who received medical care between 1 March 2020 and 31 December 2020 to best reflect the timeframe of the pandemic before the widespread availability of COVID-19 vaccinations. The study population included adult Veterans (age ≥18 years) with active utilization of VHA medical services within the past 12 months (defined by a clinical visit at a VA site within the previous 12 months of the COVID-19 test) and who had at least one available COVID-19 result tested at a VA site. Exclusion criteria was any ever prescribed use of iodine-containing medications [amiodarone, iodine-containing topical antiseptics (e.g., povidone iodine), and saturated solution of potassium iodide]. The flow of study sample selection is detailed in Figure 1; there were 47,667 COVID-19 tests with iodine contrast exposure in the past 60 days (of which 2,065 were positive) and 483,275 COVID-19 tests without iodine contrast exposure in the past 60 days (of which 30,056 were positive).

### Measurements

Iodine exposure was determined by ICD-9 and ICD-10 codes of radiologic procedures that used iodinated contrast media (Supplementary Table 1). Eligible COVID-19 tests were those measured by polymerase chain reaction (PCR) from a salivary, nasopharyngeal, or oropharyngeal source (22). It was possible for an individual to contribute data of multiple negative COVID-19 tests; however, only the first occurrence of a positive COVID-19 test was included, after which time any subsequent data were censored.

### Statistical analyses

Summary statistics (means, standard deviations, and proportions/percentages) were generated to describe the study cohort. Multivariable logistic regression models were evaluated employing generalized estimating equations were used to estimate the adjusted odds ratios (OR) and 95% confidence intervals (CI) of the associations between iodinated contrast exposure and incident COVID-19 infection. Covariates were age, sex, race/ethnicity (Hispanic, non-Hispanic White, non-Hispanic Black, non-Hispanic Asian, non-Hispanic American Indian/Alaskan Native, non-Hispanic Native Hawaiian, and Other Pacific Islander, and unknown/missing), place of residence (urban, rural, and unknown/missing), socioeconomic status (SES) (defined by CDW priority groups 1–8, where groups 1–6 = high SES and groups 7–8 = low SES, and unknown/missing), and insurance status (Medicaid, Medicare, other, and unknown/missing). These potential confounders were selected *a priori* from among those available in the CDW database under the hypothetical relationship between iodine exposure and COVID-19 infection. After fitting the models, we employed the *margins* command in STATA to calculate the adjusted prevalence of COVID-19 infection among iodine contrast exposed and unexposed groups. To evaluate the possible difference in the association by demographic characteristics, we also stratified the analyses by age (<65 years and ≥65 years), sex (male and female), and race/ethnicity (Hispanic, Non-Hispanic White, Non-Hispanic Black, and Non-Hispanic others). All analyses were performed using STATA version 17. The study is compliant with all STROBE criteria for cohort studies and was approved by the Veterans Affairs Greater Los Angeles Healthcare System Institutional Review Board.

## Results

The study sample was composed of 530,942 COVID-19 tests from 333,841 Veterans (mean ± SD age, 62.7 ± 15.2 years; 90.2% men; 61.9% non-Hispanic Whites) (Table 1). Each patient contributed a median of one COVID-19 test (range, 1–30). Overall, there were 47,667 (9.0%) COVID-19 tests in patients who had received iodinated contrast media in the past 60 days (Table 2).

**TABLE 1:** Descriptive characteristics of the study cohort (*n* = 333,841 subjects).

	No. (%)	
	Receipt of iodine ≤60 days of COVID-19 testing	No receipt of iodine ≤60 days of COVID-19 testing
Age (years), mean ± SD	66.6 ± 12.8	61.3 ± 15.7
**Sex**		
Male	32,884 (92.72)	266,085 (89.18)
Female	2,581 (7.28)	32,291 (10.82)
**Race/ethnicity**		
Hispanic	2,235 (6.30)	26,102 (8.75)
Non-Hispanic White	22,759 (64.17)	185,341 (62.12)
Non-Hispanic Black	8,337 (23.51)	66,161 (22.17)
Non-Hispanic Asian	163 (0.46)	2,340 (0.78)
Non-Hispanic American Indian/Alaskan native	208 (0.59)	2,023 (0.68)
Non-Hispanic Native Hawaiian and other Pacific Islander	205 (0.58)	2,064 (0.69)
Unknown/missing	1,558 (4.39)	14,345 (4.81)
**Place of residence**		
Urban	24,412 (68.83)	203,015 (68.04)
Rural	4,223 (11.91)	40,291 (13.50)
Unknown/missing	6,830 (19.26)	55,070 (18.46)
**SES**		
Low SES	4,241 (11.96)	33,121 (11.10)
High SES	31,216 (88.02)	264,823 (88.75)
Unknown/missing	8 (0.02)	432 (0.14)
**Insurance type**		
Medicaid	3,613 (1.21)	406 (1.14)
Medicare	94,757 (31.76)	14,978 (42.23)
Other insurance	98,022 (32.85)	10,587 (29.85)
**VA primary care encounter within the past 18 months**		
No	1,046 (2.95)	12,498 (4.19)
Yes	34,419 (97.05)	285,878 (95.81)

**TABLE 2:** Subject characteristics by COVID-19 test status (*n* = 333,841 subjects).

	No. (%)	
	COVID-19 negative	COVID-19 positive
**Sex**		
Male	270,061 (89.5)	28,908 (90.3)
Female	31,756 (10.5)	3,116 (9.7)
Age, years, mean ± SD	62.0 ± 15.4	63.5 ± 15.4
**Race/ethnicity**		
Hispanic	24,646 (8.2)	3,691 (11.5)
Non-Hispanic White	189,972 (62.9)	18,128 (56.6)
Non-Hispanic Black	66,630 (22.1)	7,868 (24.6)
Non-Hispanic Asian	2,268 (0.8)	235 (0.7)
Non-Hispanic American Indian/Alaskan native	1,989 (0.7)	242 (0.8)
Non-Hispanic Native Hawaiian and Other Pacific Islander	2,018 (0.7)	251 (0.8)
Unknown/missing	14,294 (4.7)	1,609 (5.0)
**Place of residence**		
Urban	205,037 (67.9)	22,390 (69.9)
Rural	40,312 (13.4)	4,202 (13.1)
Unknown/missing	56,468 (18.7)	5,432 (17.0)
**SES**		
Low SES	33,271 (11.0)	4,091 (12.8)
High SES	268,148 (88.8)	27,891 (87.1)
Unknown/missing	398 (0.1)	42 (0.1)
**Insurance type**		
Medicaid	3,754 (1.2)	265 (0.8)
Medicare	100,523 (33.3)	9,212 (28.8)
Other insurance	97,975 (32.5)	10,634 (33.2)
No insurance	99,565 (33.0)	11,913 (37.2)
**VA primary care encounter within the past 18 months**		
No	12,124 (4.0)	1,420 (4.4)
Yes	289,693 (96.0)	30,604 (95.6)

SES, socioeconomic status.

**FIGURE 2:**
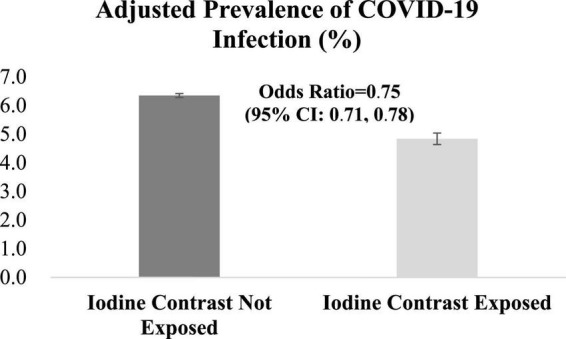
Associations between iodinated contrast exposure and risk of COVID-19 infection. *P*-value for Chi-square test statistics was <0.001.

**FIGURE 3:**
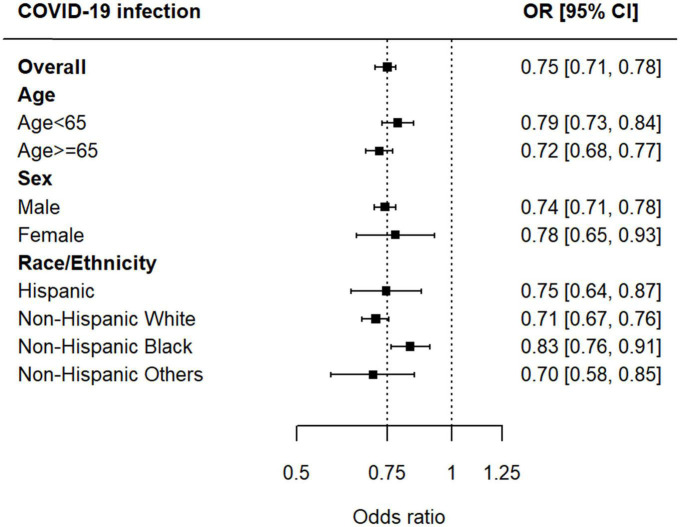
Associations between iodinated contrast exposure and risk of COVID-19 infection by age, sex, and race/ethnicity.

Prior iodine exposure was associated with a decreased risk of incident COVID-19 test positivity (OR, 0.75 95% CI, 0.71–0.78) (Figure 2). In the stratified analyses, we found no evidence of the heterogeneity in the associations between iodinated contrast use and increased risk of COVID-19 infection by age (<65 years, OR 0.79, 95% CI 0.73–0.84, *p* < 0.001; ≥65 years, OR 0.72, 95% CI 0.68–0.77, *p* < 0.001; *p* for the interaction between age and COVID-19 infection, *p* = 0.25), sex (male, OR 0.74, 95% CI 0.71–0.78, *p* < 0.001; female, OR 0.78, 95% CI 0.65–0.93, *p* < 0.001; *p* for the interaction between sex and COVID-19 infection, *p* = 0.17), and race/ethnicity [Hispanic, OR 0.75, 95% CI 0.64–0.87, *p* < 0.001; Non-Hispanic White, OR 0.71, 95% CI 0.67–0.76, *p* < 0.001; *p*-for-interaction (vs. Hispanic), *p* = 0.95; Non-Hispanic Black, OR 0.83, 95% CI 0.76–0.91, *p* < 0.001; *p*-for-interaction (vs. Hispanic), *p* = 0.06; and Non-Hispanic others (vs. Hispanic), OR 0.70, 95% CI 0.58–0.85, *p* < 0.001; *p*-for-interaction (vs. Hispanic), *p* = 0.93] (Figure 3).

## Discussion

From data obtained from the largest integrated healthcare system in the U.S., this study is the first to demonstrate the protective function of a high iodine load, as conferred by a single administration of radiologic iodinated contrast, against incident COVID-19 infection. These data suggest that judicious supplemental iodine intake may be a cheap, easily accessible, and effective public health measure to help address the pandemics due to enveloped viruses, particularly prior to the development of effective vaccines, in regions of the world where COVID-19 vaccination rates have not yet achieved herd immunity, and when mutants have escaped immune surveillance. Nevertheless, the study’s results must be carefully interpreted and not be used to support dietary and supplemental iodine use in excess of its recommended Tolerable Upper Limit (1,100 mcg/day) (5). Given the potential adverse risks of supraphysiologic iodine exposure to thyroid function, the American Thyroid Association cautions against ingestion of iodine supplements containing >500 mcg iodine per daily dose (23), recommendations that we strongly endorse.

Our findings support the biological principles of an LDH-mediated defense mechanism against SARS-CoV-2. Patel et al. reported an inhibitory constant (Ki) of ∼10 μM for HOI generated against influenza viruses *in vitro* (18); HOI has further been shown to be viricidal against SARS-CoV (24). *In vivo* experiments have demonstrated that iodine supplementation in sheep may have viricidal benefits (13, 25, 26). The LDH innate defense system and its conversion of iodine to an endogenously produced disinfectant, HOI, demonstrates that reasonable and safe iodine exposure may decrease risk of infections from disinfectant-sensitive enveloped viruses such as SARS-CoV-2. Further support of the antiviral effects of oxyhalides generated by the LDH system include the effect of excess SCN- on COVID-19 infection. SCN-, the other main substrate for LPO, is also concentrated in the saliva and bronchial secretions, generating disinfectant OSCN-. Populations exposed to excess SCN- such as cigarette smokers and consumers of inadequately prepared cassava root also appear to be protected against COVID-19 infection (27, 28). We have reported that excess iodide exposure increased iodide and HOI output in saliva with positive correlation between iodine load and changes salivary iodide levels, and salivary iodide output and salivary HOI output (28), suggesting that iodine supplementation enhances salivary viricidal HOI bioavailability which may prevent HOI-sensitive viral infections including SARS-CoV-2.

The protective function of iodine against COVID-19 infection may, in part, explain the disparate rates of SARS-CoV-2 positivity observed worldwide. The incidence of COVID-19 infections early in the pandemic were relatively wide-ranging between the U.S., Japan, and South Korea (6.4, 1.4, and 0.3%, respectively, as ascertained on 1 June 2020) (29), despite the overall higher population densities and urban crowding in many parts of Asia. Although there may be many other explanations for this variation, individuals residing in Asia have generally a much higher dietary intake of dietary iodine, in particular from kelp; the Japanese typically ingest ≥1–2 g iodine/day (30) whereas the average iodine consumption on the U.S. is approximately 150 mcg iodine/day, according to minimally adequate median urinary iodine concentrations of the general population (31).

The strengths of the present study include the use of the largest integrated healthcare system in the U.S.; the data comprehensively capture individual participant-level iodine exposure used in routine clinical settings and subsequent COVID-19 testing. Another strength is the biological plausibility of the data given independent corroborative studies reporting that potentially antiviral concentrations of salivary HOI in subjects following radiocontrast dye injection and other epidemiologic data suggesting decreased COVID-19 transmission among populations exposed to excess amounts of the halide/pseudohalide LPO substrates iodine and thiocyanate. Nonetheless, our study has the following limitations: First, given the uncertainties of accurate medical coding required for any secondary database analyses, misclassification bias is possible. In particular, there is the possibility of misclassification due to the many types of molecular (and not antigen) COVID-19 tests selected to denote active infection of SARS-CoV-2 that we sought to ascertain. Second, although we included important demographic characteristics in our model, there are some unmeasured confounders between iodine exposure and COVID-19 infection, including comorbidities and their severities. Thirdly, our results from this male-predominant VA database may not be necessarily generalizable to other populations. Future studies with measurements and adequate information on medical conditions among non-VA patients would be warranted to validate, replicate, and generalize our findings. Lastly, the iodine content of iodinated contrast media administered to participants was unavailable in our dataset. Although the use of any iodinated contrast dose confers several hundredfold the daily iodine requirements for normal thyroid hormone synthesis, additional research may also examine the potential dose-response of our findings.

This population-based assessment of the associations between iodine exposure and incident COVID-19 infection support the antimicrobial activity of iodine against a defensible infection. These data suggest that iodine can potentially protect against SARS-CoV-2, and iodine supplementation restricted to <500 mcg per daily dose (23) may represent an effective approach to decreasing the global burden of COVID-19 infection, particularly in regions where vaccinations against SARS-CoV-2 are not yet readily available, effective, or administered yet to sufficient proportions of the population.

## Data availability statement

The raw data supporting the conclusions of this article will be made available by the authors, without undue reservation.

## Ethics statement

The studies involving human participants were reviewed and approved by the VA Greater Los Angeles Healthcare System Institutional Review Board. Written informed consent for participation was not required for this study in accordance with the national legislation and the institutional requirements.

## Author contributions

AL: concept and design. MM and AL: acquisition, analysis, or interpretation of data. KT, KI, and AL: drafting of the manuscript. KT, KI, MM, JK, YA, ML, NN, JC, RE, MB, and AL: critical review of the manuscript. MM, KI, and AL: statistical analysis. All authors contributed to the article and approved the submitted version.
