# Supplementary material for: Iodine contrast exposure and incident COVID-19 infection
Source: Front Med (Lausanne). 2022 Dec 1;9:1033601. doi: 10.3389/fmed.2022.1033601 (PMC9751341; doi:10.3389/fmed.2022.1033601)
Supplement: Supplementary file 1 [file Table_1.DOCX]

**Supplemental Table 1.**  CPT and HCPCS codes used to define radiologic iodinated contrast exposure.

| **Types of Radiologic Iodinated Contrast Studies** | **Codes** |
| --- | --- |
| Iodinated CT scans and angiography | CPT: 36221, 36222, 36223, 36224, 36225, 36226, 36227, 36228, 36251, 36252, 36253, 36254, 70460, 70470, 70480, 70481, 70482, 70487, 70488, 70491, 70492, 70496, 70498, 71260, 71270, 71275, 71551, 71552, 72126, 72127, 72129, 71275, 72130, 72132, 72133, 72174, 72175, 72191, 72193, 72194, 73201, 73202, 73206, 73701, 73702, 73706, 74150, 74160, 74170, 74174, 74175, 74177, 74178, 74145, 74174, 74175, 74177, 74178, 75574, 75635, 75572, 75574, 75600, 75605, 75625, 75630, 75635, 75658, 75705, 75710, 75716, 75726, 75731, 75733, 75736, 75741, 75743, 75746, 75756, 75774 |
| Venograms | CPT: 75820, 75822, 75825, 75827, 75831, 75833, 75840, 75842, 75860, 75870, 75872, 75880 |
| Cystograms | CPT: 74455 |
| Hysterosalpingograms | CPT: 74740, 76830, 76831, 76856 |
| Urograms | CPT: 74170, 74178 |
| Any procedure using iodine as contrast agent | HCPCS: Q9958, Q9959, Q9960, Q9961, Q9962, Q9963, Q9964, Q9965, Q9966, Q9967 |
